# Vulnerability of human settlements to flood risk in the core area of Ibadan metropolis, Nigeria

**DOI:** 10.4102/jamba.v9i1.371

**Published:** 2017-11-24

**Authors:** Rafiu O. Salami, Jason K. von Meding, Helen Giggins

**Affiliations:** 1 School of Architecture and Built Environment, University of Newcastle, Australia

## Abstract

Flood disasters continue to wreak havoc on the lives of millions of people worldwide, causing death and massive economic losses. In most African cities, residents and their assets are among the most vulnerable to flood risks in the world. The nature and scale of this urban risk are changing because of the dynamic patterns of land use, unplanned growth and impacts of climate change. Flood risk is the product of the flood hazards, the vulnerability and exposure of the people and their physical environment. In order to minimise flood disaster, there is an urgent need to understand, invest in flood disaster risk reduction for resilience and to enhance disaster preparedness for an effective response as articulated in the recent Sendai Framework for Disaster Risk Reduction. This research utilises a new proposed flood vulnerability assessment framework for flood risk in a traditional community in the heart of Ibadan metropolis, in the context of their households’ exposure, susceptibility and coping capacity through a well-designed questionnaire survey. The study uses descriptive and inferential statistics techniques to provide a detailed understanding of the vulnerability profiles of the community and the levels of residents’ preparedness to mitigate the flood risk. The results of the statistical analysis show that there is a significant relationship between residents’ flood awareness and having previous flood experience, but there is no significant association between their awareness of risk and the level of preparedness for flooding. To minimise exposure and vulnerability to flood risk, we advocate effective adaptation policies to achieve disaster risk reduction and resilience on flood risk rather than focusing merely on reactive measures after disaster strikes.

## Introduction

It is widely acknowledged that human interference with the climate system has been the major cause of climate change and the observed global warming (Hansen et al. 2007; Ramanathan & Feng 2008; Rockström et al. 2009). Given the inextricable connection between climate change and development (Douglas et al. 2008), the Intergovernmental Panel on Climate Change (IPCC) warns of the possibility of an increase in the frequency and intensity of catastrophic weather events such as temperature extremes and consistent rain and windstorms (Pachauri & Reisinger 2007; Parry 2007). The continuous unstoppable rapid urbanisation, particularly in developing countries (Wong 2015), and poorly managed urban growth and land use, coupled with destructive effects of climate change, have been the dominant cause of natural and man-induced disasters such as earthquakes, cyclones, landslides, sea-level rise, tsunami, flooding and erosion among others (Hardoy, Mitlin & Satterthwaite 2013; Mitlin & Satterthwaite 2013). For instance, in African cities, hydro-meteorological hazards, including floods and droughts, are regarded as the most common of all hazards (Van Niekerk 2015; Van Niekerk & Wisner 2014).

Floods are unarguably the most common of all natural hazards (Jha, Bloch & Lamond 2012) and also affect more people than all types of natural disasters put together (emergency events database [EM-DAT] 2015). Flood disasters are responsible for over 50% of all casualties and more than 30% of global economic losses from natural disasters (Hallegatte et al. 2013). It is estimated that the average annual population of people affected by the flood is likely to increase from 1 million in 1990 to 25 million by 2050 (Sachs 2006). According to CRED’s EM-DAT, over the last two decades, floods, storms, heatwaves or other weather-related events caused 90% of all disasters the world over. Flooding alone affected 2.3 billion (56%) people worldwide (Table 1) (EM-DAT 2015), and at least 20% of the Nigerian population was affected by flood disasters (Oyekale 2013).

**TABLE 1:** Number of people affected by weather-related disasters (1995–2015).

Natural disasters	Number of people affected	Percentage of people affected
Floods	2.3 billion	56
Drought	1.1 billion	26
Storm	660 million	16
Extreme temperature	94 million	2

*Source*: EM-DAT, 2015, *The human cost of weather-related disasters, 1995–2015*, Centre for Research on the Epidemiology of Disasters, UN Office for Disaster Risk Reduction (UNODRR), pp. 1–25, Brussels, Belgium

Similarly, despite considerable advancement and technological capabilities for dealing with floods, flood disasters continue to cause severe damages even in developed countries. For instance, EM-DAT preliminary data for 2016 reveals that 301 reported disasters affecting 102 countries caused by geophysical, meteorological and hydrological hazards resulted in 7628 deaths, affected 411 million people and caused economic damages to the tune of $97 billion (EM-DAT 2016). The report indicates that 50% of all disaster events in 2016 were related to flooding, and storms represent 22% of all natural disasters reported in the same year (EM-DAT 2016). Previous studies on exposure and residents’ vulnerability to urban flooding in Ibadan and the other cities in Africa are still limited. Solutions to the problems of regular flooding remain unclear. A detailed analysis and assessment that will provide in-depth insight, which can address the threat of flooding and vulnerabilities of urban residents in Ibadan, is lacking. More diversity would be required to bridge the gap that exists, particularly when compared with advanced countries.

In recent years, the frequency and intensity of rainfall events, flash floods, acute riverine and coastal flooding have been on the increase, corresponding with more reported cases of flood disasters across the world (Vojinović 2015). It is highly important to focus on proactive measures rather than common focus on responding to the disaster. In line with Hyogo framework for action (HFA) guidelines (UNISDR 2005), even the recently adopted Sendai Framework for Disaster Risk Reduction (SFDRR) (Kelman 2015) clearly recognises the urgent need to create a holistic and robust flood risk management strategy that can effectively address the problem of urban floods. There is still little knowledge and poor understanding of specific types and causes of flooding, their probabilities of occurrence and the potential vulnerable population and/or assets, as well as areas affected particularly at the local level (Adelekan et al. 2015). Given the importance of exploring how flood risk and vulnerability are spatially distributed within urban cities in developing countries, including Nigeria, particularly at the community level, this article provides a better understanding of the nature and scale of urban residents’ vulnerability to flood risk and the level of households’ preparedness in Bere, a flood-prone and traditional core area of Ibadan.

## Understanding the flood risk

Flood risk is described by Bates and De Roo (2000), UNISDR (2009) and Birkmann (2007) as the product of flood hazards, the associated vulnerability and exposure of the people and their physical environment. According to Merz et al. (2007), flood hazard is ‘the exceedance probability of potentially damaging flood situations in a given area and within a specified period’ (p. 236). The magnitude and scale of flood damage are not only influenced by the flood’s characteristics but also depend on the vulnerability profile of a particular area (Birkmann 2007). It is also regarded as ‘the combination of the hazardous phenomenon of flooding and a vulnerable system susceptible to suffer loss’ (Eleutério 2012:2). Not all hazards automatically result in disaster, the determinant drivers that turn hazards into catastrophic events are the level of vulnerability and the degree of susceptibility of a population to disaster risk (Birkmann et al. 2013). In order to understand the concept of flood risks, a comprehensive knowledge of the human system is very important.

Flood vulnerability involves elements at risk such as the residents of a flood-prone area, a built environment or an ecosystem exposed to flood risk (Merz et al. 2007). Meanwhile, vulnerability is generally acknowledged by many researchers to consist of three components: degree of exposure, susceptibility and resilience or response capacity of a population in a particular area (Birkmann 2006; Jean-Baptiste, Kabisch & Kuhlicke 2013; Pandey, Manandhar & Kazama 2014; Wisner et al. 2004). Besides these components, vulnerable communities can be further evaluated through a variety of vulnerability determinant drivers such as physical, social, economic, environmental and political factors (Wisner et al. 2004).

A system is susceptible to floods because of its exposure, and its capacity or incapacity to be resilient, cope, recover or adapt to the extent of damage (Balica, Wright & Van der Meulen 2012). With growing evidence on cities’ flood vulnerability, most flood-related disasters are not primarily caused by natural disasters. Many scholars acknowledge that the primary determinant factors are largely attributed to human activities that involve socio-political, historical and cultural relations (Birkmann 2007; Milly et al. 2002; Seyoum et al. 2011; Vojinović 2015; Vojinović & Abbott 2012). While the lack of basic knowledge and understanding of flood risk by the people living in flood-prone areas may have contributed to ineffective decision-making, Pelling and Wisner (2012) note that poor governance and social and environmental injustice are the underlying causes of flood risk. For instance, a city with a very low quality of basic infrastructure, unplanned growth and rapid urbanisation coupled with the effects of climate change means heavy rainfall can manifest as a catastrophic flood (Baker 2012; Global Footprint Network 2012).

## Flood risk vulnerability in Ibadan

In Nigeria, like other developing countries, the impacts of flood disasters on urban residents, their housing and other assets are significant (Ravallion, Chen & Sangraula 2007) because of the influence of climatic changes, increase in demographic growth and urbanisation of poverty (UNISDR 2002, 2015; Von Meding et al. 2011). The continuous exposure of city dwellers to flood and other disaster risks are intensifying urban poverty and their vulnerability (International Federation of the Red Cross-crescent Societies [IFRC] 2010).

Ibadan is one of the metropolitan cities in sub-Saharan Africa that is facing problems of severe flooding and windstorms that are more frequent. The city’s vulnerability to flood risk is a function of the region’s exposure to natural hazards and the anthropogenic influence that contributed immensely to this risk (Bouwer 2011; Swyngedouw 2013). Flooding is a natural phenomenon. However, human factors have exacerbated the flooding menace (Ajayi et al. 2012; Douglas et al. 2008; Eguaroje et al. 2015). These factors include unplanned urban growth, construction of unregulated substandard informal settlements on the flood plain, disregard to waste management culture and lack of proper maintenance of drainage channels. Urban residents in Ibadan and most cities in developing nations are particularly vulnerable when disasters like flooding strikes because of their limited coping capacity and meagre resources to mitigate the effect. Most of the low-income households are faced with the loss of their natural, physical and social assets without hope of recovery or support from the local institutions (World Bank 2006).

Flood disaster is not a recent phenomenon in the city of Ibadan. According to several scholars, more than 16 major flood disasters of varying degree of intensity have occurred in the ancient city (Agbola et al. 2012; Eguaroje et al. 2015; Tomori 2008) and over 35 000 deaths were recorded with loss of assets worth several millions of Naira (Ajayi et al. 2012). Etuonovbe (2011) affirms that the disaster that has caused displacement and has affected Nigerians the most in history is flooding. According to Eguaroje et al. (2015), in the findings in their study on Ibadan flood vulnerability assessment, there are only 11 007 (9%) houses that are located in the less vulnerable area out of 128 182 houses in the big city. In other words, 91% of all houses in Ibadan metropolis are vulnerable to flood risk with varying degrees of susceptibility, ranging from least vulnerable (55.9%) to highly vulnerable (25%). However, there is still a limited research focusing on a better understanding of the magnitude and scale of urban exposure to flood risks and the impacts on human settlements at the micro level or communities within Ibadan metropolis. It is the focus of this study to assess the vulnerability of an indigenous community in the core area of Ibadan to flood risks, measuring the residents’ exposure, resilience and adaptive capacity using physical and socio-economic variables.

## Urban housing vulnerability and development management in African cities

Housing is known as the most affected sector in any catastrophic events. Millions of houses have been destroyed because of natural and man-induced disasters from the earthquakes, cyclones to floods, storms and fire accidents (Tipple 2005). Around 87 million homes and 130 000 schools, clinics, hospitals and education facilities were either damaged or destroyed, with floods and storms accounting for around 98% of houses damaged (EM-DAT 2015). The level of poverty, housing quality, the state of infrastructure, as well as awareness about disaster risks and households’ preparedness will determine the impact of disaster events on society and environment (Olorunfemi & Adebimpe 2008).

In the study area, there is the abundant legacy of congested and poor houses which are unfit for habitation, characterised by unhealthy neighbourhood conditions, indiscriminate dumping of wastes and inadequate infrastructure services (Coker et al. 2008). One of the key drivers of mortality during a natural disaster, like floods, is the structurally defective urban fabrics, and unregulated constructed houses, particularly in developing countries (EM-DAT 2015). Urban poor generally live in the most dangerous and unhealthy environment (Baker 2012). Many low-income households in cities are at risk of multiple hazards because of their spatially distributed substandard houses located on river flood plains and unstable hilltop and hazardous areas (Douglas et al. 2008). Like other African cities, Ibadan residents are exposed to natural hazards like flooding and windstorms as well as day-to-day hazards such as lack of access to essential services, quality housing and adequate environmental infrastructure (Adelekan et al. 2015). Most communities in the city lack basic amenities and good infrastructure facilities, in addition to the consequences of climate change which may compound their vulnerability to disaster risks.

In most cases, the urban planning policies and building codes guiding land use and/or development are not regularly updated to meet urban growth’s new direction in the developing world. The local planning authorities are ineffective and ill-equipped to enforce planning regulations and ultimately lack the capacity to oversee urban development management so as to reduce disaster risk (Parnell, Simon & Vogel 2007). For example, the local authorities that are responsible for monitoring and enforcing building codes in all the urban districts in Ibadan are incapacitated (Measham et al. 2011). They have limited resources and power to play these important roles (Dodman & Satterthwaite 2008; Satterthwaite 2011).

## Justification of the study

Globally, cities in developing countries are increasingly prone to flood risk, particularly in socio-economically deprived areas (Pelling 2011). Flooding is the most frequent and widespread disaster in the world with significant death toll and economic loss (EM-DAT 2016). Ibadan is a flood-prone city with official records of floods in 1951, 1955, 1960, 1963, 1973, 1978, 1980, 1982, 1984, 1986, 1987, 1997 and 2011 (Agbola et al. 2012; Oguntala & Oguntoyinbo 1982; Olaniran 1983; Tomori 2008). Bere, the study area, and most communities in the core area of Ibadan are vulnerable to flood risk. Besides their proximity to rivers and the topography of their location, the highly populated settlements are also characterised by low quality, deficient, dilapidated buildings, with poor structural quality, as well as the lack of basic and infrastructural facilities (Adelekan 2016; Coker et al. 2008). The effects of social vulnerability are manifested in cities of developing countries through the growth of slums where poverty is rife, and where marginalised social groups within cities exist. For instance, more than one-third of city dwellers live in slums, characterised by poorly constructed houses lacking basic facilities such as clean water and proper sanitary and drainage systems (Baker 2012; UN-Habitat 2013). This implies that most residents have a high chance of exposure to disaster risk and vulnerability to hazards such as flooding, and they have limited resources to cope or adapt if a disaster strikes. Therefore, an in-depth assessment is necessary to unveil the root causes of urban settlements’ vulnerability to flood hazards in the first place and to better understand the factors that determine inequitable exposure to flood vulnerability in African cities.

## Overview of the study area

**FIGURE 1:**
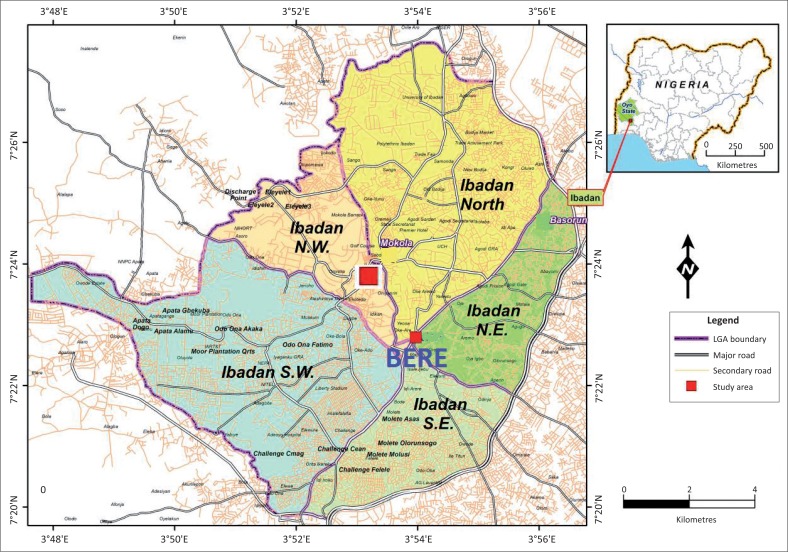
Map of Ibadan metropolis showing Bere location in the core area of the city (2016).

The city of Ibadan is the administrative headquarter of the old western region of Nigeria and now Oyo state’s capital. It (Figure 1) is the third largest metropolitan area, by population, in Nigeria. It has a long history of flood events and is recognised as a flood-prone area with many floods recorded since 1902 (Tomori 2008), but only officially recorded from 1951 (Agbola et al. 2012). A series of unprecedented floods have killed hundreds of people and destroyed residents’ properties worth millions of Naira. For instance, more than 600 hundred people lost their lives in flood disasters that occurred on 31 August 1980 and 26 August 2011 (Agbola et al. 2012). While the heaviest rainfall recorded (274 mm in August 1980) was during a single flood episode, the next heaviest was 258 mm in August 1963. The devastating flood in August 2011 (187.5 mm) affected the city’s public assets, urban settlements and agricultural land, causing domestic and economic damages worth around 30 billion Naira (Agbola et al. 2012).

As the metropolitan area continues to attract rapid population growth, many urban residents are forced to live in floodplains and other hazardous areas. The poor disposal of domestic sewage and industrial waste contributed significantly to flood incidences because of blockages in river channels in most places in Ibadan (Coker et al. 2008). The urban growth and urbanisation witnessed in the city are widely attributed to the influx of rural–urban migrants, because of the availability of economic opportunities such as the presence of industries, an array of institutions and infrastructural services (Owoeye & Ogundiran 2014). This unprecedented development overwhelmed the little resources available and inadequately maintained services (Salami et al. 2015).

**FIGURE 2:**
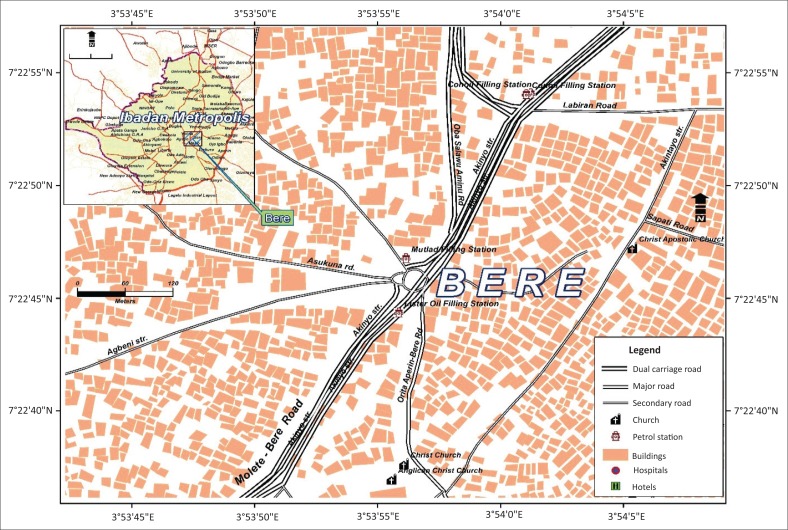
Map of Bere community at the core of the city (2016).

The research was carried out in Bere, a typical inner core area of Ibadan that represents the pinnacle of pre-colonial urban development in Nigeria with a high density of closely built houses and people (Adelekan 2012). Bere, the study area, is one of the communities categorised under the traditional core of Ibadan. The community is in the form of informal settlement dating from the pre-colonial era and is characterised by closely built low-quality houses like compounds, closely connected, with no provision of road access and basic services such as potable water and a sewerage system. The highly populated settlement is characterised by low-quality buildings and is predominantly inhabited by the indigenous people of Ibadan (EnyinnayaEluwa, Siong & Abayomi 2012). The community lacks basic and infrastructural facilities such as clean water, roads, sanitary facilities and drainage (Ipingbemi 2010). Many of the buildings are deficient, dilapidated, have poor structural quality and are vulnerable to flooding. By all standards as described by UN-Habitat (2003) and Fabiyi (2004), Bere reflects a housing environment that is in poor conditions with an unhealthy spatial distribution (Figure 2). The residents are slum dwellers and low-income earners. In Mabogunje’s words, 49 years ago, concerning the core areas of Ibadan, ‘slum dwellings characterised by no identifiable sanitation facilities, housing in mud, physical deterioration and the highest population density area of the town’ (Mabogunje 1968:233).

The Bere community’s spatial pattern was suited to the socio-economic conditions of the pre-colonial times. For decades, most indigenous people inhabited these areas, and other low-income earners continued to occupy these traditional core areas with the highest density of residential houses compared to other residential zones in Ibadan. These densely populated slums consist of about 26254 housing units (Adelekan 2016) that lack drainage systems and adequate sanitation facilities (Coker et al. 2008).

## Research method and data

The research is based on a quantitative method using primary data drawn from questionnaire administration **i**n Bere, a traditional community located at the heart of the metropolitan city of Ibadan, Nigeria. The Bere and other indigenous communities in the core of Ibadan are categorised as high-density areas, one of the three major classifications of residential land-use characteristics in urban areas of Ibadan (Adigun 2013; Afon & Faniran 2013). Secondary data were sourced from academic journals, textbooks and government documents. Quantitative methods entail a series of numerical data which are measurable by instruments for statistical analysis (Creswell & Clark 2007). A quantitative instrument such as the questionnaire is a versatile tool used to obtain information about perceptions of certain issues from respondents (Wisker 2007). An assessment of urban settlements’ vulnerability utilising this form of inquiry requires testing theories by a deductive approach relying on identified indicators (Birkmann 2006; Kuhlicke et al. 2011). The study employed a systematic random sampling technique to select the dwelling units for questionnaire administration using the door-to-door approach. This sampling technique makes the task easier because of its simplicity and the assurance of achieving an evenly sampled population compared to simple random sampling. The selection of households was chosen using a uniform interval of a minimum of five houses, after the first element (household and/or dwelling unit) has been randomly placed along one side of the road, as appropriate to the size of the communities.

Out of 250 questionnaires administered to household heads, 156 fully completed ones were returned. The response rate was 62.4%, and the remaining 37.6% were either not available at home when researchers returned twice to collect the questionnaires or the target household head declined to answer the questionnaire. Data were then entered into Statistical Package for the Social Science (SPSS) and carefully analysed using descriptive and inferential statistical techniques. Some statistical analyses were generated to report results in the form of charts, percentages in tables and graphs. The researcher explored inferential statistical techniques utilising analysis of variance (ANOVA) (Kruskal–Wallis Test in SSPS), correlation (Spearman’s rho) and chi-square (Cramer’s V) to further examine the relationships between the flood risk perceptual variables and socio-economic variables (Robson 2011).

**FIGURE 3:**
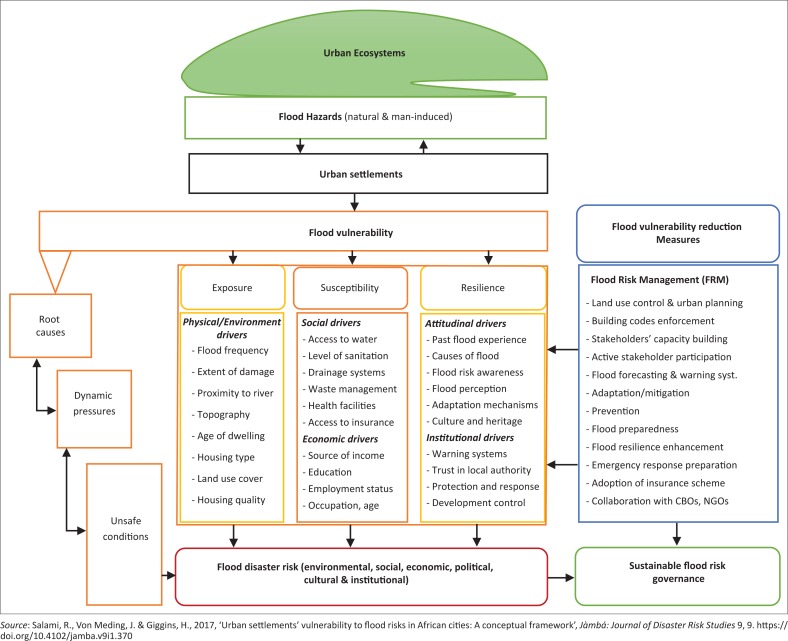
Proposed analytical vulnerability assessment framework for Ibadan City.

The well-designed questionnaire was based on our recently developed flood vulnerability framework (Figure 3) and review of existing literature to have a deep understanding of the levels of the vulnerability to flood risk and impacts on urban settlements and their residents taking into cognisance the devastating flood disaster of August 2011. The survey was also prepared to assess households’ socio-economic and demographic characteristics, housing quality, physical and/or structural conditions of the housing stock, as well as evaluation of basic and/or infrastructural services, respondents’ environmental conditions, the flood risk awareness, experience, preparedness, coping capacity and mitigation measures. The Human Research Ethics Committee (HREC) issued ethical approval in June 2015, and appropriate informed consents had also been approved by all participants in this research. The data collected were coded, analysed and interpreted using SPSS.

Given the fact that vulnerability is multidimensional, unequal, scale dependent and dynamic (Vogel & O’Brien 2004), and flood vulnerability frameworks that embrace holistic approaches in the context of African cities are still limited (UNISDR 2011), Figure 3 presents a flood vulnerability assessment framework that exemplifies how urban settlements in Ibadan interact with natural and man-induced hazards, which could cause disasters (such as urban floods), that are likely to affect vulnerable urban poor residents (Salami, Von Meding & Giggins 2017). Their flood vulnerability is the result of the dynamic interaction (between biophysical and human factors) which Birkmann (2006) describes as three progressions of vulnerability – root causes, dynamic pressures and unsafe conditions. This framework adopts these three stages of vulnerability (Figure 3) as applicable to typical urban settlements in African cities. For instance, the underlying causes of flood vulnerability in African cities are triggered by differential access to livelihood income, tenure security and bad governance, among others (Baker 2012).

## Results and discussions

The outcomes of the questionnaire survey with the head of households on human settlements’ vulnerability to flood risks in Bere, an indigenous community in the core area of Ibadan metropolis, provide a detailed understanding of impacts of the exposure, sensitivity and adaptive capacity of the residents. This section presents analysis and discussions of the data collected through the administration of questionnaires to the studied community. These include demographic and socio-economic characteristics of the sampled households, as well as physical, structural and infrastructural conditions of the urban settlements. In addition, the vulnerability profiles of the households regarding flood experience, perceptions, preparedness and adaptive coping mechanisms for future flood risk were also analysed.

### Demographic and socio-economic profiles of respondents

The study administered 250 questionnaires to household heads based on door-to-door survey in the studied communities between September and October 2015, with a response rate of 62.4%. The remaining 37.6% of the sampled population was not available at home when the researchers returned twice to collect the questionnaires, or the target household head declined to answer the survey. The findings of this study from the returned questionnaires (*n* = 156) as shown in Table 2 reveal that 69% of respondents were male and 31% were female. Forty-two per cent (66) of the respondents sampled were between the age group of 41 and 50 years. While the age group above 50 years accounts for 25% of the total respondents, 18% fall in age group between 21 and 30 years. This is consistent with the average household size of Ibadan city according to 1991 National Population Census reports (Tomori 2008). According to Buckle, Mars and Smale (2000), family size can influence urban poor’s vulnerability and their coping capacity in a disaster. An overcrowded household can stress the occupants’ coping and have a serious effect during an emergency response to a disaster (King & MacGregor 2000).

**TABLE 2:** Demographic profile and composition of households’ survey participants.

The study area	Bere	
	*n*	%
Number of questionnaires distributed	250	-
Number of questionnaires responded	156	-
Percentage responded	0	62.4
**Gender**		
Male	108	69
Female	48	31
**Age**		
18–20	2	1.3
21–30	28	17.9
31–40	21	13.5
41–50	66	42.3
51–60	26	16.7
61 above	13	8.3
Missing	0	0

Table 3 summarises the socio-economic features of the 156 households sampled in this study. Within the socio-economic profiles of the respondents, attributes considered were household size, education, occupation and monthly income. The table reveals that the majority of the sampled population (64%) have between 4 and 6 persons as the size of their households. Sixteen per cent of all respondents comprised between 1 and 3 occupants in their households. This is consistent with the average household size of Ibadan city according to 1991 National Population Census reports (Tomori 2008), and it is similar to the findings of an empirical study on household vulnerability to food poverty in Ibadan metropolis (Odusina 2013).

**TABLE 3:** Socio-economic, physical/structural and basic/infrastructural characteristics of households in Bere community.

Parameters	Frequency (*n* = 156)	Percentage (**%**)
**Socio-economic characteristics**		
**Households’ size**		
01-Mar	25	16
04-Jun	99	63.5
07-Sep	24	15.4
10+	8	5.1
**Level of education**		
No formal education	14	9
Primary/secondary	133	85
ND/NCE/HND/Bsc	9	6
**Occupation**		
Artisan	58	37.2
Farmer	5	3.2
Student	9	5.8
Civil servant	2	1.3
Professional	2	1.3
Trader	72	46.2
Other	8	5.1
**Monthly Income(Naira)**		
< 20 000	116	74.4
20 001–40 000	27	17.3
40 001–60 000	3	1.9
60 001 and above	1	0.6
None	9	5.8
**The physical/structural characteristics**		
**Age of the building**		
01-Mar	1	0.6
04-Jun	1	0.6
07-Sep	6	3.8
Ten years and above	148	95
**Wall construction materials**		
Mud	114	73.1
Cement block	15	9.6
Sun-dried brick	6	3.8
Bamboo with mud	21	13.5
**Structural conditions**		
Needs minor repair	54	34.6
Needs major repair	89	57.1
In good condition	12	7.7
Others	1	0.6
**Basic/infrastructural facilities conditions of the neighbourhood**		
**Access to water**		
Borehole	12	7.7
Well	48	30.8
Outside my yard (< 200 m)	86	55.1
Outside my yard (> 200 m)	9	5.8
Through water tanker	1	0.6

The level of education of most respondents (85%) was either primary or secondary education as their highest academic qualification. While 9% of the total respondents had no formal education, 6% of the sampled population had post-secondary education. The literacy level of the sampled population greatly influenced the nature of the occupation and the status of their livelihoods. The majority of the respondents were engaged in informal economy, ranging from trading and artisanship to farming, and 1% each were professionals and public servants. The findings of the study also revealed that 74% of the respondents earned 20 000 Naira (the equivalent of 80 USD) or less in a month. Only 17% of the sampled population earned between 21 000 and 40 000 Naira, while 6% of the total respondents had no job. Many scholars have argued that communities with low human capital households (such as low income and poor education) face higher exposure to flood risk and lower level of flood preparedness (Brouwer et al. 2007; Pelling 2011; Ward & Shively 2017).

### Impacts of exposure and susceptibility to flood hazards

**FIGURE 4:**
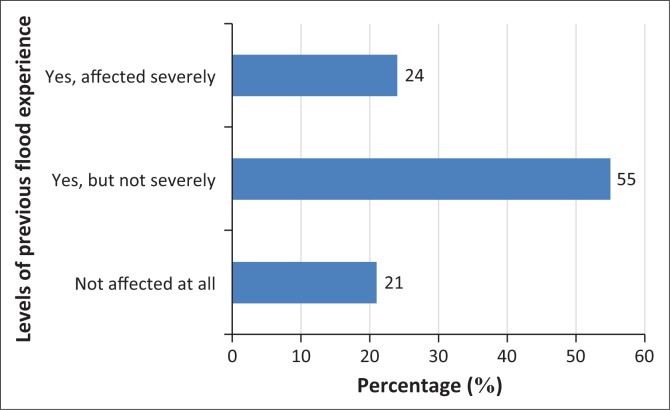
Percentage of respondents’ flood events experience.

Given decades of flood events history recorded in the indigenous city with the recent August 2011 flood event (Agbola et al. 2012), the study investigates the impacts of the residents’ exposure and susceptibility to flood risks. It employs questionnaire survey to assess issues related to flood experience such as whether the respondents have been previously ‘severely affected’, or ‘affected but not severe’ or ‘not affected at all’. The results of the study in Figure 4 indicate that 55% of all the respondents were not severely affected, 24% were severely affected and 21% were not at all affected by previous flood disasters. In order words, around 79% of the sampled population experienced flood disaster with different degrees of severity. For example, the August 26 Ibadan flood disaster was one of the most catastrophic in the city’s flood history, causing serious casualties with more than 100 people losing their lives and resulting in economic losses of more than 30 billion Naira (Agbola et al. 2012).

The characteristics of the respondents’ houses (Table 3) indicate an array of variables such as the age of the residential houses, the construction materials and structural conditions to basic and infrastructural facilities. The survey of the community shows that 95% of the sampled respondents’ houses were old with deteriorating urban spatial environmental condition and years of constructions, categorised under the age group of 10 years and above. Each household sampled was asked to rate their satisfaction concerning the structural condition of their residential houses. The quality of housing structure and its location are significant indicators categorised under physical vulnerability drivers in Ibadan metropolis (Table 3). The structural conditions of the houses were deplorable with varying degrees of disrepairs as more than half of the sampled settlements (57.1%) in Bere (high density, low-income area) needed varying major repairs of their buildings to be structurally fit, and only 7.7% houses were in good condition. Housing structure serves as a predictor variable to assess housing quality in the study sites, and a determinant of occupants’ level of exposure and vulnerability to flood hazards (Rumbach & Shirgaokar 2017).

Similarly, overall assessment of the quality of construction materials in the surveyed areas reveals that 73% of the buildings were built with weak wall construction materials (mud), and less than 5% utilised cement blocks which are regarded as one of the best construction materials. According to Adelekan et al. (2015), indigenous communities such as Bere are categorised as one of the old informal settlements in Ibadan, characterised by the lack of basic amenities such as clean water, good drainage system and sanitation services. This implies that the majority of the residents are slum dwellers (UN-Habitat 2015) with a high level of flood exposure and vulnerability. Inadequate housing, unhealthy living and lack of good nutrition are regarded as the causal factor for vulnerability (Birkmann & Wella 2016).

In addition, this study indicates that the sampled populations’ perception of the causes of flood disasters in Ibadan is majorly centred on the blockage of natural and artificial waterways, rain, building in flood plains and improper planning of the city. Table 4 shows how respondents’ perceptions on the causes of flooding in the study varies from one another. Blockage of waterways and high intensity of rainfall were considered as major reasons for frequent flooding in the area by 53% and 44% of respondents, respectively. Regarding the impact of the previous floods on the respondents’ houses and properties, 37% of the sampled population claimed that they experienced destruction of houses, while about 45% experienced loss of property as a result of flood hazards and about 17% of the respondents had no economic loss attributed to the previous flooding in the study area.

**TABLE 4:** Flood risk perception and adaptation strategies.

Parameters	Frequency (*n* = 156)	Percentage (**%**)
**Flood-related damage before**		
Not affected at all	33	21
Yes, but not severely	86	55
Yes, severely	37	24
**Causes of floods**		
Heavy rainfall	68	43.6
Blockage of waterways	83	53.2
Building on flood liable plains	4	2.6
Improper planning and poor land use	1	0.6
**Economic loss**		
Destruction of property	58	37.2
Destruction of houses	71	45.5
Loss of lives	1	0.6
None	26	16.7
**Coping/adaptive strategies**		
Forced migration	25	16
Maintenance of house	20	12.8
Use of quality construction materials	10	6.4
Support from family/friends	6	3.9
Prayers	82	52.6
Insurance	0	0
Government support	3	1.9
Indebtedness through borrowing	10	6.4
**Total**	**156**	**100**

### Flood risk awareness, preparedness, adaptive coping and mitigation measures

**FIGURE 5:**
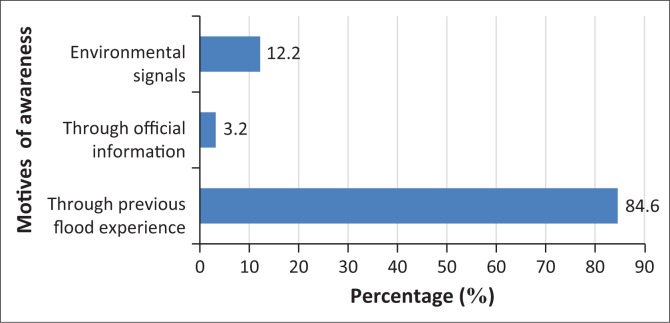
Percentage of respondents’ motive of awareness of flood risk.

As indicated in Figure 5, the authors investigate whether the respondents were aware of the risks attributed to flooding before the occurrence through previous flood experience or other means. Most of the respondents (85%) claim that the motive of awareness was through their previous flood experience and another 12% of the household heads ascribe their awareness to environmental signals. The percentage of respondents that were informed through government official information was just 3%. A chi-square test confirmed a significant relationship between flood risk awareness and past flood experience (*x*^2^ = 58.965, *P* = 0.000). The result of this study agrees with findings in studies by Parker, Priest and Mccarthy (2011), Grothmann and Reusswig (2006) and Burningham, Fielding and Thrush (2008).

**FIGURE 6:**
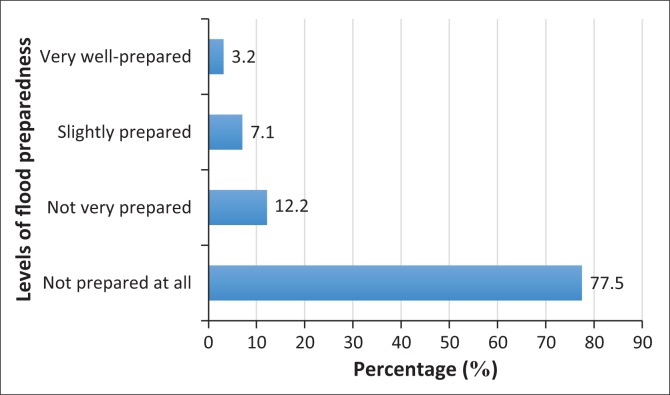
Percentage of respondents’ rank of level of preparedness.

**FIGURE 7:**
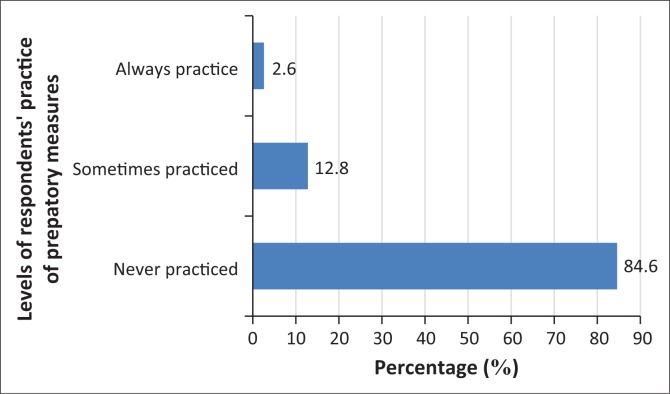
Percentage of respondents’ practice of preparatory measures for flood risk.

Despite the overwhelming flood risk awareness among residents, the results of the study concerning the respondents’ level of preparedness to face a future flood risk (Figure 6) reveal that 78% of the sampled population opined that they were ‘not prepared at all’, while 12% and 7% of the respondents claimed they were ‘not very prepared’ and ‘slightly prepared’, respectively. Only 3% of the respondents indicated that they were well-prepared to face future risks of floods. In another similar question posed to the respondents (Figure 7), ‘Do you practice any preparedness measures for flood-risk mitigation?’, the results indicate that most of the respondents (85%) never practiced any preparedness measures, around 13% sometimes engaged in some measures to mitigate flood risk and around 3% ‘always practiced’ risk mitigation measures. The authors further utilise chi-square tests to examine the association of risk awareness with levels of preparedness which were found to be (*x*^2^ = 6.00, *P* = 0.423). This implies that there is no significant relationship between the two variables. Also, the researchers conduct a statistical test utilising correlation (Spearman’s rho) to further examine the association of risk awareness with levels of preparedness. The result (*r* = -0.097, *P* > 0.05) shows that there is no significant relationship between the two variables.

**FIGURE 8:**
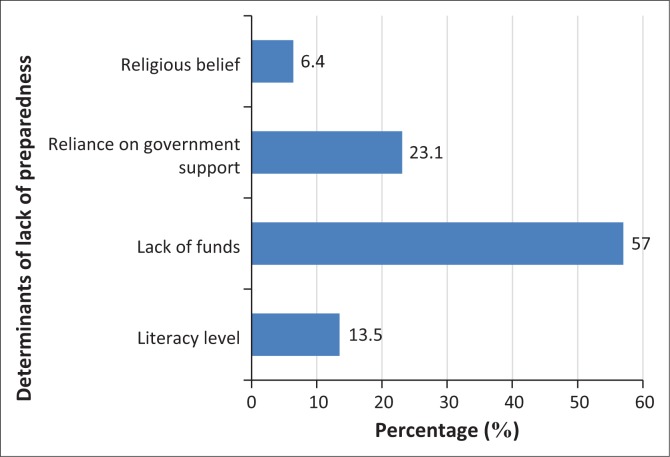
Percentage of respondents’ practice of preparedness for flood mitigation measure.

According to the findings of the study, the main reasons provided by the respondents for their failure to adopt preparatory measures to mitigate future flood risk were ‘lack of funds’ and ‘reliance on government’ to provide structural devices. Around 57% of the total respondents (Figure 8) claim lack of funds was responsible for the inability to engage in preparatory measures, while 23% believed that local authority or government at different levels has the statutory obligation to put preventive measures in place to minimise the future flood risks. In order to further verify the assumption, the authors statistically examine the influence of respondents’ socio-economic profile on their decisions, utilising chi-square test to clarify whether there is a statistically significant relationship between the livelihood patterns and level of preparedness (Table 5). The results reveal that there is a strong relationship between the level of income of respondents and level of preparatory measures to flood risk in the study area. In line with this study, Donahue, Eckel and Wilson (2013) and Baker (2011) found a significant association between households’ income and the level of preparedness of residents related to their response to natural disasters.

**TABLE 5:** Results of cross tabulation between level of preparedness and socio-economic variables.

Socio-economic variables	Level of preparedness
Income	(0.015)*
Education	−0.415
Occupation	−0.24

Chi-square (Cramer’s V) value in bracket.

* Significant correlation

**FIGURE 9:**
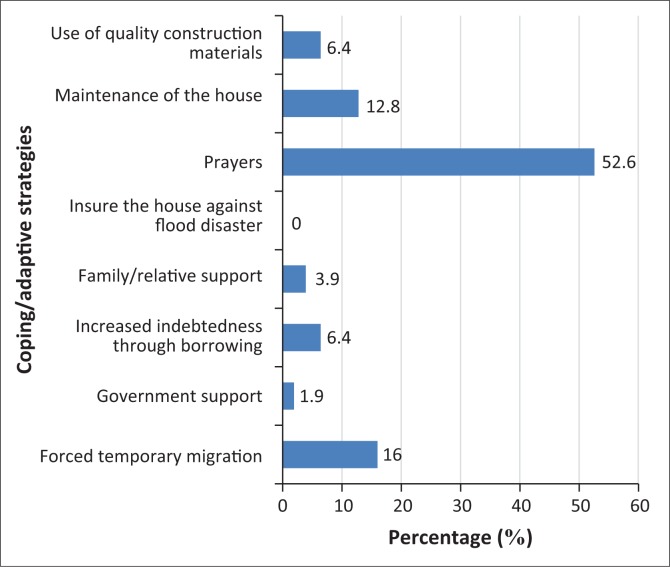
Percentage of respondents’ adaptive coping mechanisms.

The adaptive coping strategies adopted by the respondents in the study area according to the findings of the study (Figure 9) are forced temporary migration, maintenance of buildings, engagement in prayers, the use of quality construction materials and increased indebtedness through borrowing. More than half of all the respondents (53%) consider prayers, a religious belief, as the most common adaptation strategy. The consideration of prayer as the best option for the adaptive measure is consistent with the research findings of Adelekan (2012) and Haque and Blair (1992) on populations in developing countries who are vulnerable to wind hazards and tropical cyclone, respectively. None of the respondents contemplates insurance as flood mitigation measure, while 16% of the sampled population adopt forced temporary migration as an option and around 13% consider regular maintenance of their houses as adaptive coping mechanisms.

**FIGURE 10:**
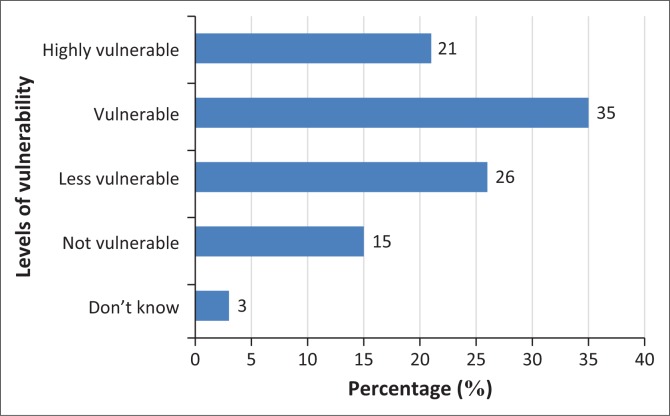
Percentage of respondents’ rank of vulnerability to risks attributed to floods.

In addition, the analysis of the respondents’ self-assessment in Figure 10 demonstrates their level of vulnerability to flood risk, which was based on a Likert scale of 5-point rating (where 1 denoted ‘don’t know’ and 5 denoted ‘highly vulnerable’). The results show that 21% of the sampled population were ‘highly vulnerable’, and 35% and 26% of the respondents considered themselves ‘vulnerable’ and ‘less vulnerable’, respectively. According to Few (2003), coping capacity is one of the determinant factors of the levels of vulnerability of a household or community. Yohe and Tol (2002) and the World Health Organization (2002) affirm that adaptive capacity depends on the profiles of individuals, households or community in the context of their social and human capital. In other words, human settlements or households such as the sampled population with a low level of income and few productive assets are most likely to have low resilience to flooding.

## Ethical considerations

An approval letter from the HREC at the University of Newcastle, Australia was received before the commencement of data collection. The ethical clearance was approved in June 2015 with the HREC protocol no is H-2015-0112.

## Conclusion and recommendations

This study provides an evaluation of a human settlement’s vulnerability to flooding risk in Bere, an indigenous community situated at the heart of Ibadan metropolis, Nigeria. This was achieved through an assessment of the magnitude and scale of the residents’ exposure, susceptibility and the impacts on the spatial dispersion of the area. The study utilises a case study approach in the flood-prone urban local community using households’ questionnaire survey to elicit vital information that relates to the residents’ awareness, the level of preparedness, preparatory measures adopted and the rating of their vulnerability to risks attributed to flooding. The questions asked also included respondents’ physical, environmental, socio-economic and institutional variables.

The unprecedented dynamics behind an increase in urbanisation and unplanned urban growth have made developing countries particularly African cities vulnerable to multidimensional disaster risks. The impacts of climate change and variability have been acknowledged as a key factor that will further exacerbate the urban risks (IFRC 2010; IPCC 2013). Residents of most cities are forced to live in illegal settlements located in dangerous places such as flood-prone areas because of the low level of income, lack of access to few resources and lack of good governance (Hardoy, Mitlin & Satterthwaite 2001).

The findings emerging from the research are interesting and also surprising. The results of this study indicate that there is a statistically significant association between resident’s risk awareness and previous flood experience. However, there is no significant relationship between residents’ flood risk awareness and level of preparatory measures to future risk. In other words, the higher level of residents’ awareness of flood risk may not automatically turn to a higher level of preparedness to mitigate the risk. This study outcome is consistent with several studies (Bradford et al. 2012; Harries & Penning-Rowsell 2011; Jóhannesdóttir & Gísladóttir 2010; Karanci, Aksit & Dirik 2005; Scolobig, De Marchi & Borga 2012; Siegrist & Gutscher 2006). However, contrary to this view, there are scientifically proven submissions that there is a strong relationship between risk awareness and level of preparedness (Ruin et al. 2007; Shidawara 1999; Vinh Hung, Shaw & Kobayashi 2007).

In order to resolve the weak relationships within risk perception factors and preparatory measures so as to create an effective persuasive risk communication, Duval and Mulilis propose the person-relative-to-event (PrE) model. The PrE model suggests two assessments that are related to adaptive behaviour – evaluation of experience in the past event and personal resources. The model further affirms that if the assessed previous event severity was higher than assessed personal adaptive coping resources, then adaptive behaviour may not be likely to happen (Duval & Mulilis 1999). In the same vein, Wachinger et al. (2013) suggest that three possible interventions will provide a better understanding of the reasons for the complex relationship between two mentioned variables – risk perception factors and preparatory measures. Firstly, to appraise households’ event experience and motivation; secondly, to assess trust and responsibility of government or agency; and the third is related to residents’ personal coping resources capability and conditions.

Given the above, the authors of this study further explore the effects of residents’ socio-economic variables such as income, education and occupation on their levels of preparedness to flood risk utilising chi-square tests to examine whether there are any significant relationships among the variables. The results (Table 5) show that there is a statistically significant relationship between levels of income of respondents and levels of preparedness for future flood risk. The findings are consistent with recent studies by Donahue et al. (2013), and Baker (2011) also found a significant association between households’ income and the level of preparedness related to response to the hurricane.

Most importantly, Lavigne et al. (2008) affirm that most residents in developing countries strongly acknowledged that the battle for day-to-day livelihood is far more important than engaging in preparatory mechanisms for future hazards. In support of this assumption, Figure 8 in this study indicates that ‘lack of funds’ was the major factor militating against the residents of Bere to engage in preparatory mechanisms to serve as mitigation for a future flood event. Also, Figure 10 shows residents’ self-assessment (82%) of levels of vulnerability with varying degrees (from less to highly vulnerable). Based on all these salient arguments, it implies that the residents’ insufficient means of livelihoods coupled with the lack of human and social capital are responsible for their low level of preparedness which will amount to low community resilience and increased vulnerability to flood risk despite their high level of risk awareness.

The study recommends that the government at various levels have important roles to play in preparing the vulnerable communities for future flood hazards which have been predicted to increase its frequency and intensity (IPCC 2013). It is important to seek for effective policies that will improve households’ means of sustenance, community adaptive coping resources and development of a sustainable risk communication management tool to enhance people’s capability. These policies will boost residents’ ability to adopt their mitigation measures so as to achieve a resilient human settlement.
